# Effect of graphene oxide flakes size and number of layers on photocatalytic hydrogen production

**DOI:** 10.1038/s41598-021-95464-y

**Published:** 2021-08-05

**Authors:** Ewelina Gacka, Łukasz Majchrzycki, Bronisław Marciniak, Anna Lewandowska-Andralojc

**Affiliations:** 1 Faculty of Chemistry, Adam Mickiewicz University, Uniwersytetu Poznanskiego 8, 61-614 Poznan, Poland; 2 Center for Advanced Technology, Adam Mickiewicz University, Uniwersytetu Poznanskiego 10, 61-614 Poznan, Poland

**Keywords:** Chemistry, Materials science

## Abstract

The present study explored the correlation between the photocatalytic activity toward hydrogen production of the graphene-based materials and graphene oxide (GO) morphology. In this work we applied the technique based on the combination of time-dependent sonication and iterative centrifugation cascades, which were designed to achieve nanosheets size and the number of layers selection. First such obtained GO dispersions were characterized by atomic force microscopy (AFM), scanning electron microscopy (SEM) and optical spectroscopy. Those combined measurements showed that the intensity of the π-π peak at 230 nm seems to be very sensitive to the number of layers of nanosheets. Next, GO dispersions were used to establish influence of the size and the number of layers of GO flakes on the photocatalytic hydrogen production in the photocatalytic system, containing eosin Y as a sensitizer, triethanolamine as a sacrificial electron donor, and CoSO_4_ as precatalyst. The H_2_ production efficiency varied by a factor of 3.7 for GO dispersions sonicated for various amount of time. Interestingly it was found that too long ultrasound treatment had negative impact on the GO enhancement of hydrogen production which was related to the fragmentation of GO flakes. The photocatalytic system produced the highest amount of H_2_ when graphene oxide occurs as monolayers and efficiency becomes lower with the decrease of GO sheets size. Our results demonstrate the importance of optimizing the size and the number of layers of the GO flakes prior to preparation of GO-based materials.

## Introduction

Visible-light-driven water splitting is a long-standing goal for solar energy conversion. The known systems often suffer from the low photo-to-energy conversion efficiency that is related to poor electron transport between the photosensitizer/semiconductor and the catalyst. Most of the research, that involves systems in which the light harvester and the catalyst are not chemically linked and thus interaction between the components is exclusively controlled by diffusion. A novel strategy to enhance efficient charge separation and transport in photocatalytic H_2_ production systems appeared with the discovery of graphene^1,2^. The composites photocatalyst that includes graphene could have improved charge separation which results in better photocatalytic activity. Moreover, graphene due to its unique two-dimensional structure could act as a support that increases the specific surface of the material. However, graphene is a hydrophobic material what limits its application in water splitting process. Therefore, its two hydrophilic derivatives: graphene oxide (GO) and reduced graphene oxide (RGO) were found to be more suitable for application in photocatalytic water splitting system.

Much effort has been recently applied to design and fabricate multifunctional materials based on GO and RGO^2,3^. In the past 10 years, graphene-based materials were explored by a number of research groups and the concept of incorporating graphene type materials in the photocatalytic systems have been proven valid^2–12^. The first example of the use of graphene for photocatalytic hydrogen production was the system composed of eosin Y (EY), RGO with Pt nanoparticles dispersed on its surface^13^. The largest apparent quantum yield (AQE) of 9.3% was achieved under 520 nm irradiation using triethanolamine (TEOA) as a sacrificial reagent^13^. More recently, a few graphene-based systems that employ photosensitizers and catalysts (CoSnO_x_, NiS_x_, MoS_2_) that are both derived from earth-abundant materials were also reported for photocatalytic hydrogen production^9,11,14^. Yuan et al. presented enhanced photocatalytic activity toward hydrogen generation in a non-noble metal system for photocatalytic H_2_ generation that combined Zn(II)-5,10,15,20-tetrakis(4-N-methylpyridyl) porphyrin and RGO decorated with MoS_2_ as the catalyst^14^. Enhancement of photocatalytic hydrogen evolution was attributed to the facilitation of charge separation in the presence of graphene. Despite proof-of-concept studies on the application of graphene-based materials in photocatalysis, there are still some challenges to be addressed, which are thought to greatly inhibit practical applications. Surprisingly, despite the fact that it is well accepted that graphene-based materials has the potential to boost the efficiency of photocatalytic hydrogen systems, the link between morphological properties of GO or RGO (flakes size and the number of layers) and photocatalytic performances has not been explored yet.

However, a polydispersity of graphene flakes has brought scientist attention as it presents important issues in many applications since the properties of graphene are inextricably linked to its structure^15–19^. It was reported previously that many applications require controlled nanosheet properties of materials^20–23^. For example, counter electrode in the dye-sensitized solar cell require small nanosheets^23^ whereas mechanical reinforcement of composites demands large ones^21^. Lyons et al. presented that smaller flakes result in more junctions and so lower conductivity^24^. More precisely, graphene can stack into a thick multilayer structure via van der Waals force decreasing significantly the material’s specific surface area and conductivity.

We are unaware of any deliberate works in the literature on the control of the morphology parameters of GO that was applied in the hybrid materials for photocatalytic hydrogen production. Since there is no prior study that focuses on the GO size and the number of layers selection it is currently unclear whether those parameters actually matter in photocatalytic hydrogen production. Therefore, a systematic exploration of the effect of the GO size and the number of layers is highly required to provide a reasonable correction for optimizing the photocatalytic hydrogen production efficiency. With this work, we aim at filling this gap. Most graphene-oxide dispersions contain large variations in the number of layers, lateral area, and shape of the graphene oxide flakes. Here we applied the technique based on the combination of time-dependent sonication and iterative centrifugation cascades, which were designed to achieve nanosheets size and the number of layers selection. Such obtained dispersions were used to establish a correlation between the photocatalytic activity of the graphene-based materials and GO morphology. Our results clearly demonstrated that both GO size and the number of layers influences the efficiency of the photocatalytic hydrogen production, while the effect of the number of layers was much more profound.

## Experimental

### Materials

Eosin Y and triethanolamine were purchased from Sigma Aldrich, CoSO_4_ was purchased from Alfa Aesar and graphene oxide (GO-powder < 35 mesh, C/O atomic ratio = 2.5–2.6) was purchased from Abalonyx. All experiments were performed with one batch of GO that ensured constant elemental composition. Solutions were prepared with ultrapure water (18 MΩ cm).

### Sonication treatment of GO dispersions

A total of 90 mg of GO powder were sonicated for various time in 30 mL of ultrapure water using a sonic bath (Bandelin, Sonorex Super RK 103 H, 560 W). The dispersion was sonicated under ice-cooling in order to avoid heating effects. The dispersion after various sonication time and dilution were characterized by optical spectroscopy and atomic force microscopy.

### Preparation of size fractionated GO dispersions

The dispersion of GO after sonication with the high content of monolayers determined by atomic force microscopy (AFM) referred to as stock dispersion was used for nanosheets size selection. The size selection was obtained by means of controlled centrifugation with sequentially increasing rotation speeds using stock GO dispersion. The dispersion was centrifuged in a MPW-352R centrifuge equipped with a fixed-angle rotor Nalgene 11469 (radius of rotor equals 90 mm). In the standard primary cascade, stock GO suspension was subjected to centrifugation at 4 krpm (100 min). The sediment was collected while the supernatant was centrifuged at 8 krpm (100 min). Again, the sediment was collected and the supernatant subjected to centrifugation at higher speeds 12 krpm (100 min). The concentration of nanomaterial in each fraction was determined by weighing the sediment after its drying overnight at 60 °C.

### Photocatalytic reaction

The photochemical reaction has been performed according to procedure described elsewhere^25^. In brief, in a photochemical reaction 3 mL of an aqueous solution of TEOA (0.2 M) and a certain amount of EY, GO and CoSO_4_ were placed in a quartz 1 cm × 1 cm cuvette. The pH of the solution was 10.8. The mixture was then degassed by bubbling argon through it for 30 min. Then the solution was stirred continuously and exposed to irradiation from a Broadband Halogen Fiber Optic Illuminator (Thorlabs' OSL2 High-Intensity Fiber Light Source with a 150 W halogen lamp). The generated H_2_ was monitored by a gas chromatograph (GC Agilent 7890B) equipped with a thermal conductivity detector. The experiments were repeated independently four times using each time newly prepared GO suspensions.

### Experimental apparatus

Extinction spectra of the GO dispersions were measured using a two-beam spectrometer Cary 100 UV–Vis scanning from 800 to 200 nm with 1 nm step. It should be noted that the extinction (Ext) is a combination of both the absorption (Abs) and scattering (Sca) where Ext(λ) = Abs(λ) + Sca(λ). The morphology of the GO flakes were analyzed using atomic force microscopy (AFM) Agilent 5500. The AFM imaging was realized using soft tapping mode by All-In-One Al cantilever (Budget Sensors) probe C, with a nominal force constant 7.4 N m^−1^ and resonant frequency 150 kHz on the typical scan frequency of 0.2 Hz. For such analysis, the 0.1 mg mL^−1^ GO water dispersions were highly diluted in the ratio of approximately 50 µL:1 mL, deposited on freshly cleaved mica and left at room temperature for at least 24 h. The morphology of graphene oxide flakes was also examined by scanning electron microscopy (SEM) Quanta 250 FEG, FEI. Four water dispersions of GO (0.01 mg mL^−1^) with different sonication time were drop casted on silicon wafer substrate and left at room temperature to dry. The measurements were performed in a high vacuum using accelerating voltage 2 kV.

## Results

### Sonication treatment of GO nanosheets

The raw GO sample used in this study were synthesized by a proprietary modified Hummers method and purchased from Abalonyx. The formed GO can stack into thick multilayer structure via van der Waals force decreasing significantly the material’s specific surface area and conductivity which in turn may influence on the photocatalytic performance. A sonochemical approach is widely used as a routine protocol for GO preparation prior to assembly in hybrid materials for photocatalytic hydrogen production. It was reported previously that ultrasonic treatment influences the adsorption properties of GO^26,27^.

**Figure 1:**
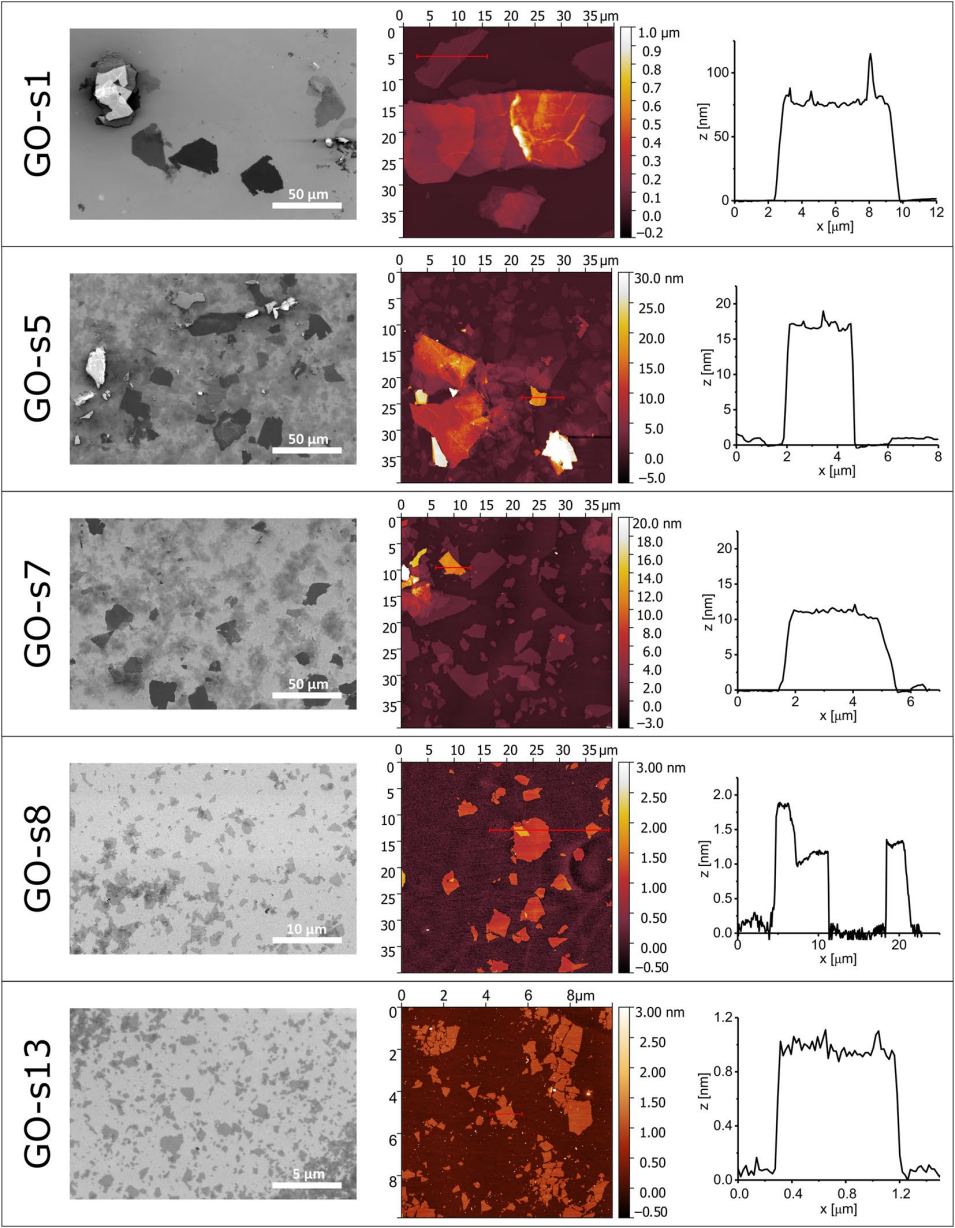
SEM images (left) and AFM images (middle) with corresponding height profiles (right) of graphene oxide flakes suspensions denoted as: GO-s1, GO-s5, GO-s7, GO-s8, GO-s13.

In this work, we investigated the effects of ultrasound sonication on graphite oxide aqueous suspensions by monitoring its structural and optical properties as a function of exposure time to the ultrasonic treatment. Martinez et al.^28^ studied the effects of ultrasounds, found that the number of layers in the GO sheets can decrease from more than 30 layers to less than 5 layers after 20 min of the ultrasound treatment. Here the variation in sonication time (1–80 min) of GO dispersion leads to obtain the suspensions, denoted as GO-s1–GO-s13, where GO-s1 was sonicated for just 1 min and GO-s13 was sonicated for 80 min. The resultant ultrasound-selected GO dispersions were characterized by atomic force microscopy (AFM) and scanning electron microscopy (SEM) (Fig. 1). What’s more, the excessive sonication of the GO dispersion can also lead to the fragmentation of GO flakes or partial reduction^26,27,29^. Thus, size-selection of the GO flakes was determined independently by cascade centrifugation (vide infra).

To characterize the morphology of graphene oxide flakes, we used two techniques of imaging: atomic force microscopy (AFM) and scanning electron microscopy (SEM). The AFM is suitable for measuring the number of layers of the flakes but during one scan only a small area of the sample is embraced. If the sample is no homogeneous, drawing conclusions based on limited number of images can be inaccurate. Thus, we used additionally SEM measurements which enabled us to observe the whole area in a reasonable time. The combination of AFM and SEM analysis provided more overall insight into the influence of the GO sonication process onto its delamination and fragmentation. Figure 1 presents the most representative images of the GO flakes from suspensions prepared with increasing time of sonication. Based on combination of the SEM and AFM results we can distinguish several structures of graphene oxide by their size and the number of layers. The first one, stacks composed of large flakes are in shiny, light gray colour (SEM) and their number of layers can reach hundreds (AFM). The large, very dark gray flakes are multilayer sheets but built of just a few layers. The smallest, light gray flakes are monolayers of graphene oxide which favour to conglomerate together during drying up drop of the sample. Based on the analysis of both AFM and SEM results we can state that the GO-s1 suspension consisted of stacks and multilayer GO flakes. Next, the GO-s5 is a mixture of smaller stacks, multilayers and monolayers, while the GO-s7 consist mostly of small multilayers and monolayer flakes. Finally, GO-s8 suspension consists mainly of monolayers. Based on these results we can conclude that initial sonication of graphene oxide breaks down the stacks which lead to the mixture of multilayers with a wide variety of flakes height down to the monolayers. Further sonication causes gradual destruction of monolayers by reducing their area (Fig. 1, GO-s13). As GO is highly sensitive for the reduction even at relatively low temperatures of 130–150°C^30,31^ we verify the apparent height of monolayered GO flakes by the AFM. As the apparent height of monolayered GO flakes remains 1.0 nm after each sonication stage this indicates that the reduction process does not occur upon sonication since for reduced graphene sheet the height of monolayered flake is expected to be reduced by 0.2 ÷ 0.8 nm^32,33^.

**Figure 2:**
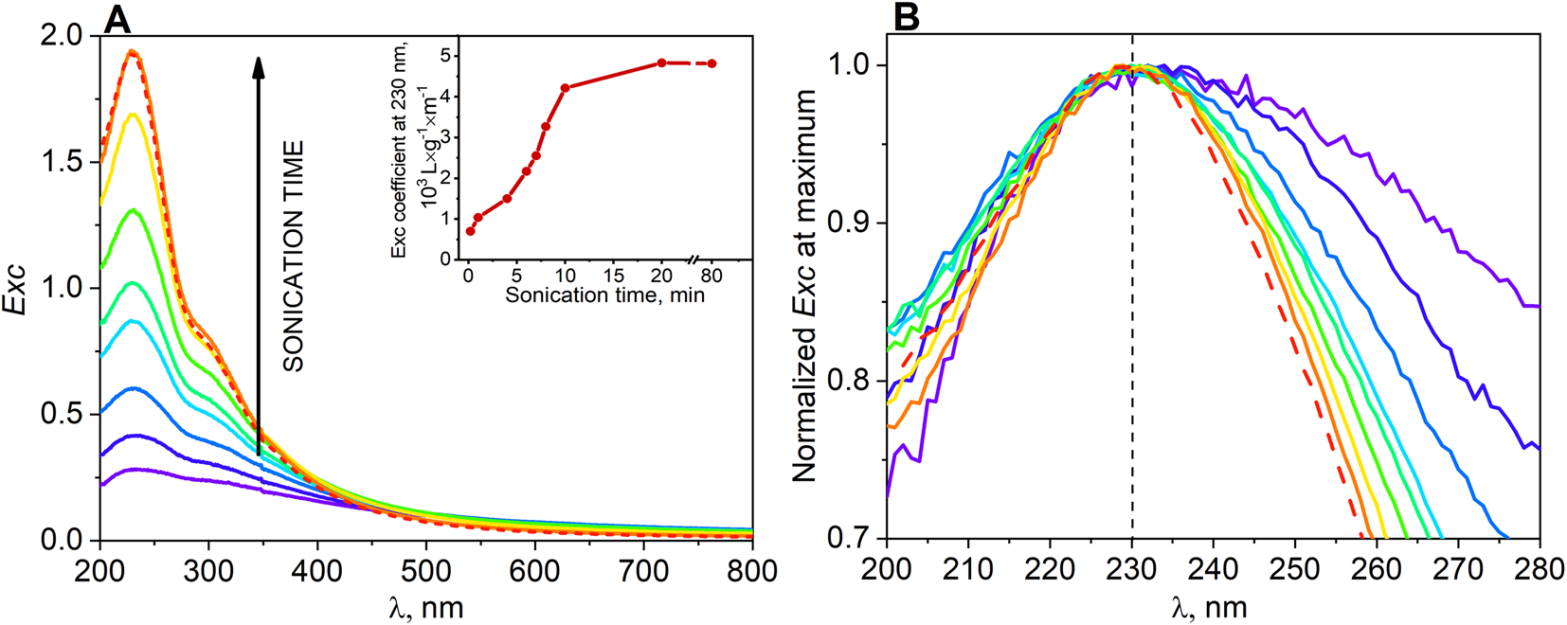
(**a**) Optical spectroscopic characterization of GO dispersions (0.04 mg mL^−1^) as a function of sonication time (1–80 min). Inset: extinction coefficient of GO dispersion at 230 nm as a function of sonication time (**b**) normalized at 230 nm optical absorption of GO dispersions (GO-s1 to GO-s13) for various sonication time.

While AFM is a reliable technique for the evaluation of number of layers of nanosheet, such a method is time-consuming and expensive, making it unsuitable for routine batch monitoring. Quantification of the number of layers of graphene oxide nanosheet via optical spectroscopy could facilitate preparation of GO dispersion with high monolayer content. Therefore, we combined AFM with optical spectroscopy in order to establish the link between the GO morphology properties and its extinction coefficients. Optical absorption spectroscopy of graphene flakes suspension produced by high-yield liquid phase exfoliation was extensively employed by Coleman’s group^4,34–36^. It is known that the lateral size distribution, the mean number of layers per flake and the functional groups on graphene and graphene derivatives are all important factors influencing the extinction coefficient^26,37,38^. As shown in Fig. 2a, extinction spectra of liquid-exfoliated GO, displayed the characteristic peak at 230 nm that is due to the C = C bond in an aromatic ring and the broad shoulder peak at 300 nm that can be assigned to C = O. There were no observed 230 nm peak shifts for all sonicated samples, that would indicate the partial reduction of the GO and restoration of the electronic configuration (Fig. 2b)^37^. In our previous study mild reduction of GO by ascorbic acid was accompanied by the significant red-shift of the peak from 230 to 259 nm^39^. Contrary to peak position that remained unchanged, the intensity of the peak at 230 nm of GO samples increased remarkably with the increase of sonication time due to efficient exfoliation of GO sheets, as observed in AFM measurements, which created more particles in the suspension and absorb more light from the excitation. In addition, the intensity of the extinction spectra of GO dispersion decreased slightly with the increase of sonication time in the region 400–800 nm which might be attributed to the decrease of the light scattering by GO samples that are exfoliated more efficiently.

It was reasonable to assume that optical absorption < 400 nm overwhelms light scattering for our flakes in such a low-concentration (0.04 mg mL^−1^). Thus the measured extinction is equal to the GO absorbance. The measured extinction can be converted to an absorbance coefficient, ε, using the Beer-Lambert law, *Exc* = *ε c l*, where *c* is the GO concentration and *l* is the cell length (1 cm). It was clear from Fig. 2a that the absorption coefficient of GO sample depends strongly on the sonication treatment of GO. The absorption coefficient of GO dispersion (0.04 mg mL^−1^) at 230 nm varied from 700 L g^−1^ m^−1^ for GO-s1 suspension that was sonicated for just 1 min to 4830 L g^−1^ m^−1^ after 20 min of sonication (GO-s8) (inset in Fig. 2a). During sonication, the GO aggregates were gradually destroyed up to the point where mainly monolayered flakes were present in suspension as determined by AFM measurements (Fig. 1, GO-s8). Based on AFM analyses (Fig. 1, GO-s8) it was determined that the GO sample that exhibited maximum absorption coefficient at 230 nm (GO-s8) existed mainly as monolayers but the presence of 2, 3 or even more layers in the sample cannot be discarded. Interestingly prolong bath sonication (additional 60 min) of the GO-s8 did not result in any further measurable changes in the optical spectra (GO-s13) (Fig. 2a, dashed line). It indicated that variation in the GO number of layers has a much more profound impact on the optical properties of GO than the GO flakes size since it was shown by SEM measurements that GO-s13 has decreased the size of GO flakes in comparison to GO-s8 sample (see Fig. 1). It is in agreement with previous reports on the decrease of GO flakes size upon long sonication^26,27,29^.

As demonstrated by combined optical spectroscopy and AFM/SEM measurements, the intensity of the π-π peak at 230 nm seems to be very sensitive to the nanosheet number of layers. Therefore we could correlate the number of layers in GO flakes with absorption coefficient at 230 nm. The number of layers in GO flakes evolve during the progressive sonication from the thick aggregates with hundreds of nanometers in thick down to fully monolayer. Concurrently, the absorption coefficient ε increases during the sonication increases. This implies the proportional correlation between the dispersion excitation coefficient and the amount of GO layers. Thus, the sonication induces the evolution of ε from ε_230nm_ = 700 L g^−1^ m^−1^ for heavier stacks composed of thick flakes (GO-s1), through ε_230nm_ = 2553 L g^−1^ m^−1^ for a mixture of monolayered and few-layered flakes (GO-s5) up to ε_230nm_ = 4830 L g^−1^ m^−1^ for fully monolayered GO dispersion (GO-s8). From UV–vis spectroscopic studies, it can be inferred that the peak at 230 nm of GO is attributed to the *π*-π* plasmon peak. It is was reported that the change of UV–vis absorption intensity at 230 nm with the number of GO layers is caused by a conjugative effect related to chromophore aggregation that influences this π-π* plasmon peak^40^.

### Size selection of GO nanosheets

Another morphology parameter of GO that may influence on photocatalytic activity is sheet size. The randomness of chemical cutting and various accessibilities of carbon on the lateral plane towards oxidants using this method yielded GO sheets with different characteristics. Therefore, to test the impact of the flakes size of GO on photocatalytic activity we have performed the cascade centrifugation in order to get size-selected GO dispersions. It was reported that for controlled centrifugation of graphene dispersions, the average lateral flake size decreases as the centrifugation rate (rpm) is increased^34–36^. Graphene oxide area sorting by means of density gradient centrifugation has been achieved using nano-GO sheets functionalized with polyethyleneglycol with the intention to obtain ultrasmall flakes for cellular imaging and drug delivery^4,34,41^.

According to the Abalonyx product specification, the average lateral size of GO is about 1 µm with a rather wide size distribution. We start with a dispersion of liquid-exfoliated nanosheets, obtained by sonication (20 min) of GO powder in an aqueous solution. Sonication time has been chosen so that number of monolayers in GO dispersion was maximized. This “stock” dispersion contained nanosheets with a broad distribution of sizes. The stock was then centrifuged at a speed (4 krpm) and the sediment collected. This sediment contained the largest nanosheets and we refer to this sample as GO-4 krpm. The supernatant produced during the 4 krpm centrifugation contained all but the largest nanosheets. It can be centrifuged at a higher rate (here 8 krpm) to give a sediment with smaller nanosheets, which we label GO-8 krpm. The associated supernatant was centrifuged again at higher rate 12 krpm. The sediment was labeled GO-12 krpm and the resulting supernatant was referred GO-residual. After each step, the sediment contained smaller and smaller nanosheets, resulting in effective size selection. Size-selected dispersions were prepared by re-dispersing the collected sediments in water after subsequently increasing centrifugation speeds.

**Figure 3:**
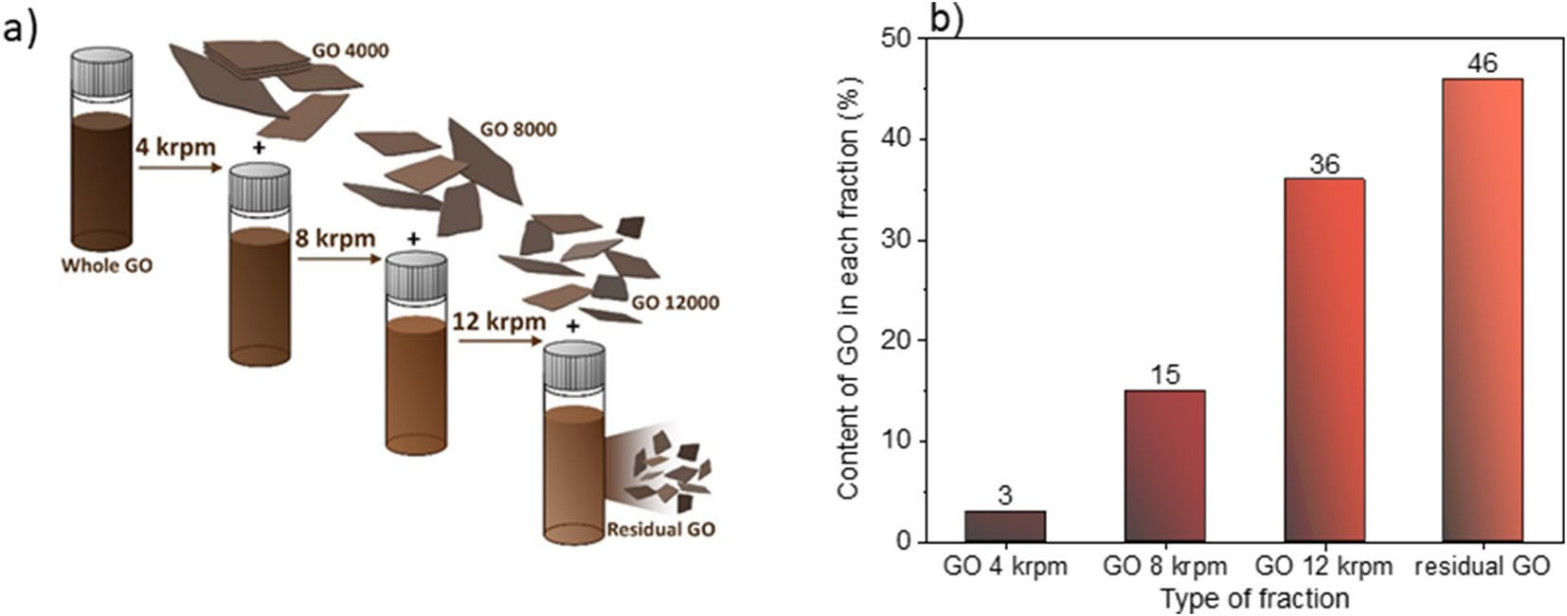
(**a**) Schematic description of the basic of centrifugation cascade employed in this study. (**b**) Percentage content of GO in each fraction in “stock” GO dispersion.

The whole process is illustrated in Fig. 3a. The content of GO in each fraction was determined by optical spectroscopy and cross-checked by drying and weighing the solid (Fig. 3b). Based on the Fig. 3b it is clear that the “stock” dispersion of GO contains as much as 46% of the GO-residual fraction. Importantly, the cascade centrifugation can be designed according to the desired outcome. Here, we wanted to produce a range of dispersions with varying nanosheet sizes in order to verify our hypothesis that the GO sheet size matters for photocatalytic hydrogen production.

**Figure 4:**
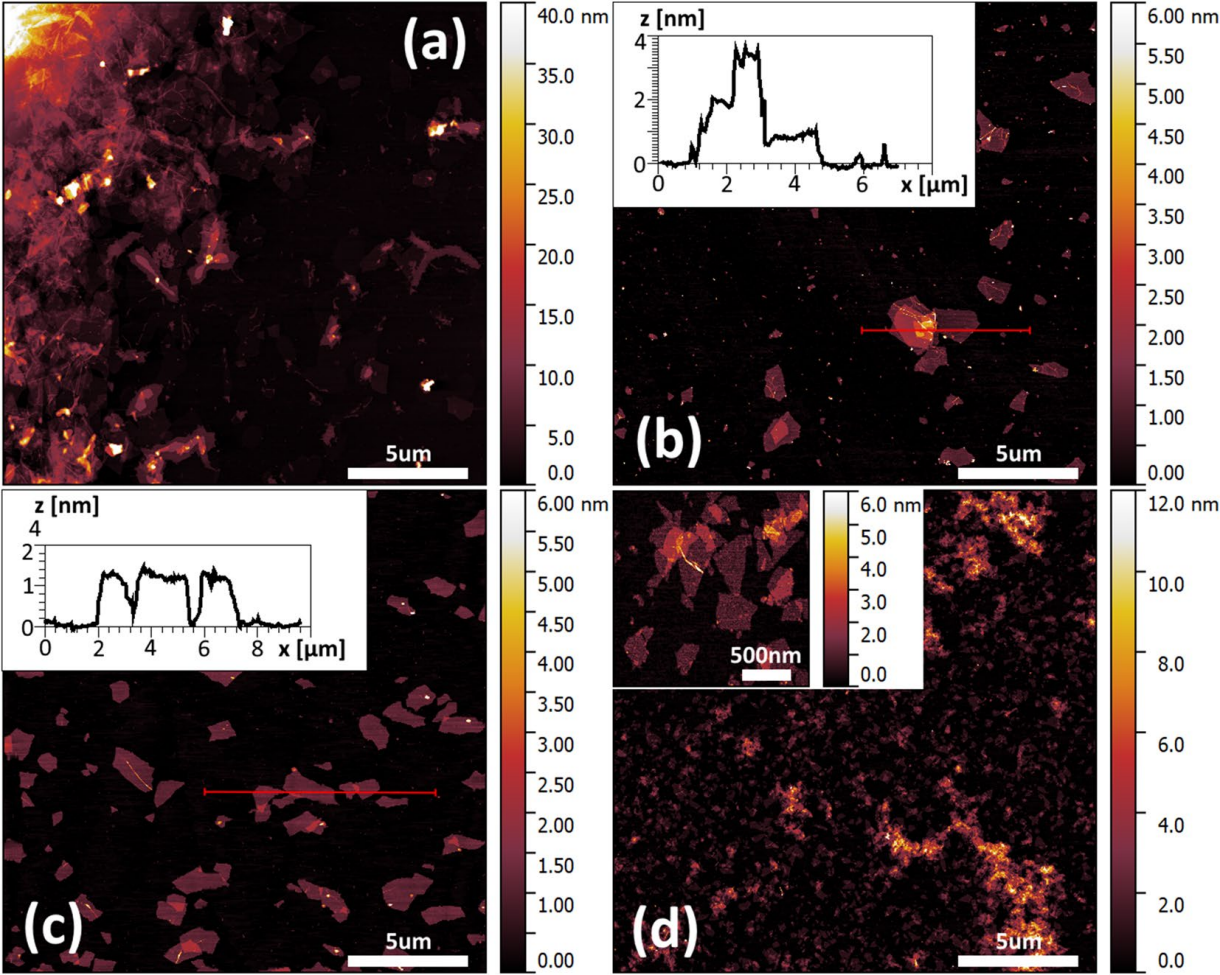
Atomic force microscopy images of graphene oxide precipitate after 4 krpm centrifugation with the presence of aggregates (**a**), the same after mild sonication (**b**), precipitate after 8 krpm centrifugation (**c**) and supernatant after 12 krpm centrifugation (**d**). Insets of (**b**) and (**c**) show the cross-section through the marked region of graphene oxide flakes, inset of (**d**) show higher magnification of GO flakes.

We have characterized the nanosheets collected in each fraction (precipitate after 4 krpm centrifugation, precipitate after 8 krpm centrifugation and supernatant after 12 krpm centrifugation (GO-residual) microscopically using atomic force microscopy (AFM) with typical images displayed in Figs. 4 a-d. In Fig. 4a, b and d the typical AFM topography of the above-mentioned fractions were shown. The GO-4 krpm fraction consisted mainly of aggregated GO flakes, while GO-8 krpm and GO-residual fractions contained mainly monolayered flakes. The “stock” GO dispersion contained only 3% of the GO-4 krpm fraction which confirm that 20 min of sonication was sufficient to obtain dispersions with high content of GO monolayers. To allow for the flakes size analysis of GO-4 krpm fraction, the mild sonication process was conducted. Resulting dispersion consists mainly of the single GO flakes, as it was shown in Fig. 4b. The apparent height of each GO flake at mica was approximately 1.0 nm as it was shown at cross-sections in insets of Fig. 4b and c. which correspond to monolayered flakes^42^.

**Figure 5:**
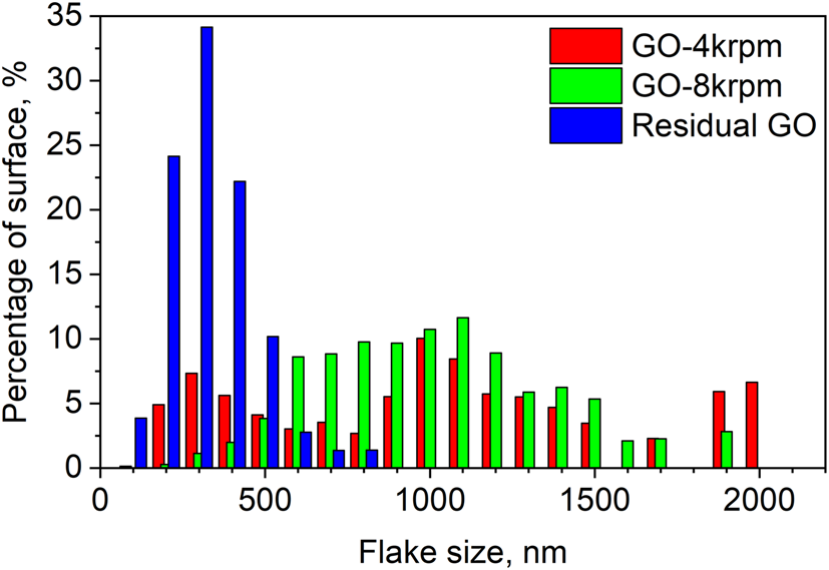
Histogram of three fractions of graphene oxide flakes from Fig. 4b, c and d.

To identify the GO size-selection of the cascade centrifugation process we performed extended statistical analysis of AFM images based on hundreds of GO flakes from each of fraction. The resulting histogram is shown in Fig. 5. The percentage intensities in size distributions were calculated to reflect the share of GO flake size in the total area of the material. These histograms show as predicted the reduction in nanosheet size as the centrifugation rates are increased. The GO-4 krpm fraction, containing mainly the aggregated GO, exhibits the wide range of flakes size up to 2000 nm in diameter with two weakly noticeable maxima near 300 nm and 1000 nm in diameter. The fraction of GO-8 krpm is dominated by the flakes in the size of 1000 nm with a slightly broader distribution, while the GO-residual fraction consists mostly of flakes in diameter of 300 nm with a very narrow size distribution.

### Photocatalytic hydrogen production

**Figure 6:**
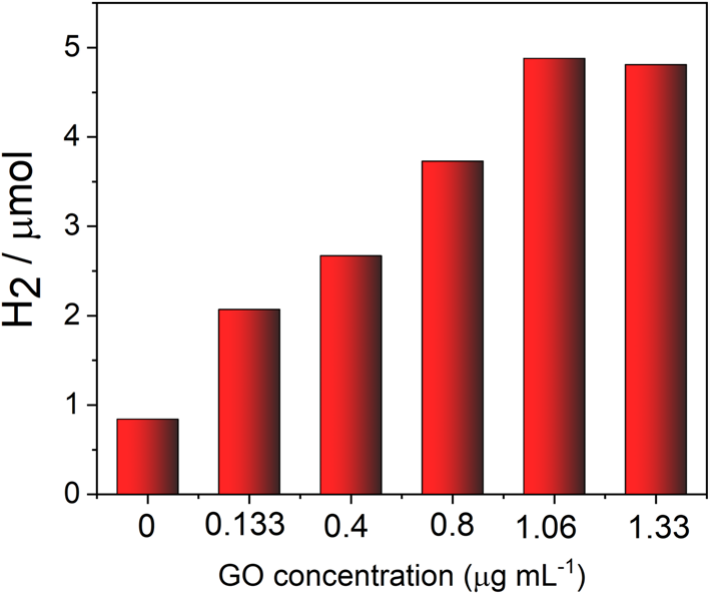
The amount of H_2_ evolved under visible light (λ > 400 nm) irradiation on EY-GO-Co^2+^ system as a function of GO concentration. Reaction conditions: EY (1 mM), TEOA (0.2 M), CoSO_4_ (2 × 10^−4^ M), pH = 10.8.

To determine whether the morphology (size and number of layers) of the GO dispersion affects photocatalytic hydrogen production, a simple non-noble metal based system was constructed. The photocatalytic hydrogen evolution was studied under visible irradiation by using triethanolamine (TEOA) as a sacrificial donor and eosin Y (EY) as a photosensitizer. CoSO_4_ salt was added to serve as a precatalyst in the reaction system, and GO was added to act as an electron mediator. This system was chosen to explore the influence of the GO morphology on photocatalytic efficiency as it was found previously that the addition of GO to these components increases the efficiency of the hydrogen production^25,43^. Wang et al. have demonstrated based on TEM, ICP-MS, and XPS measurements that Co^2+^ ions after irradiation in the presence of GO transform into Co metal nanoparticles surrounded by Co^2+^ species^43^. In addition our previous mechanistic studies have shown the existence of a stable charge-separation state after the addition of GO to the system of EY and triethanolamine what explains the role of graphene in the improvement of photocatalytic efficiency in the Eosin Y-based systems^25^. In the absence of GO, the H_2_ production from EY and CoSO_4_ system was 0.84 μmol after 1 h of irradiation. No significant amounts of hydrogen were detected in the absence of either irradiation or the photosensitizer EY suggesting that the visible light activity comes from the EY sensitization. Also no measurable amount of hydrogen was detected for the GO alone. Initially “stock” dispersion of GO-s8 (GO powder after 20 min of bath sonication) was used in order to establish the optimum GO concentration for the H_2_ production. The results of these experiments are shown in Fig. 6. With the addition of GO, even at low concentrations, the amount of hydrogen evolution showed an increase and reached a maximum of 4.88 µmol at GO concentration as low as 1.06 µg mL^−1^, which is 5.8 times larger than that of the system without GO (Fig. 6). Further increase in the concentration of GO did not result in an increase in the amount of hydrogen generated. This can be explained by the light shielding effect of graphene.

**Figure 7:**
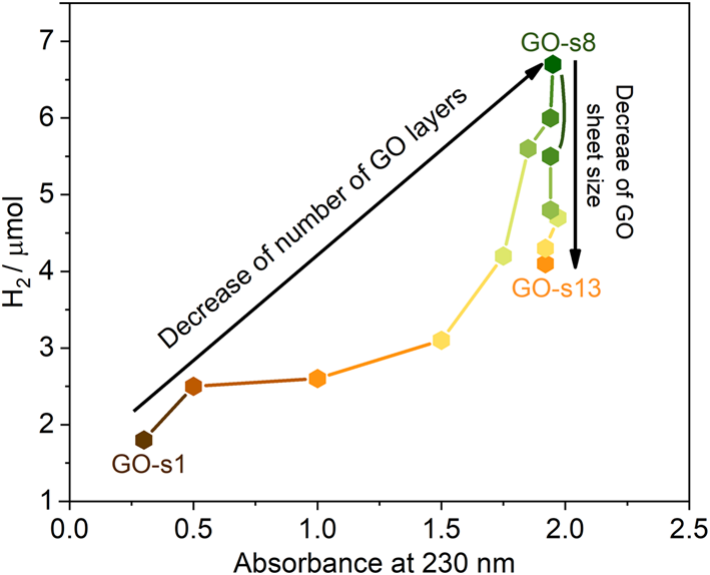
Amount of H_2_ evolved under visible light irradiation on EY–GO–Co^2+^ system with various GO suspensions with increased time of sonication. Reaction conditions: EY (1 mM), GO (1.06 µg mL^−1^), TEOA (0.2 M), CoSO_4_ (2 × 10^−4^ M), pH = 10.8.

We have performed systematic exploration of the effect of the sonication time of GO on the photocatalytic hydrogen production efficiency since the sonication process is a standard step in preparation GO solutions prior to assemblies of GO in the photocatalytic systems. In all photocatalytic tests that were performed to establish the effect of GO flakes size and number of layers on hydrogen production concentration of all components were kept constant, including GO what ensured identical conditions in all experiments. GO samples treated for various sonication times were firstly prepared and characterized (by UV–vis spectroscopy and AFM) and subsequently tested in photocatalytic hydrogen production systems. As it was shown by AFM the number of layers in GO flakes of the GO sample is correlated with the absorbance at 230 nm. The increase of absorption coefficient at 230 nm (ε_230nm_) is related to the increase of the content of monolayered flakes. The photocatalytic hydrogen generation was explored for eight samples of GO suspensions (GO-s1–GO-s8) with gradually increase of ε_230nm_ (from 700 L g^−1^ m^−1^ to 4830 L g^−1^ m^−1^) and additionally for five samples of GO (G-s9–GO-s13) with extended sonication process (total sonication time from 30–80 min) of GO suspension with ε_230nm_ = 4830 L g^−1^ m^−1^ (Fig. 2). In addition, photocatalytic H_2_ production was evaluated for GO sample referred as the sonication-free GO solution which was prepared through simple dilution of the GO powder using magnetic stirrer. Interestingly for this GO sample no increase in the hydrogen production was detected in comparison to the analogue system without GO. As presented in Fig. 7, the increase of the hydrogen production with the increase of the ε_230nm_ of the GO sample added to the system is obvious. The hydrogen production efficiency reached the maximum for the GO-s8 sample with ε_230nm_ = 4830 L g^−1^ m^−1^. According to the AFM measurements, this sample GO exists mainly as the monolayers. Based on the obtained results it is clear that efficient exfoliation of the GO dispersion is crucial for obtaining significant enhancement of the hydrogen production in the presence of GO. The H_2_ production efficiency varies by a factor of 3.7 between GO-s1 (ε_230nm_ = 700 L g^−1^ m^−1^) and GO-s8 (ε_230nm_ = 4830 L g^−1^ m^−1^) samples. However, more interestingly, the hydrogen production for systems with the addition GO samples that underwent prolong ultrasound treatment decreased. The efficiency of photocatalytic reaction decreased by 18% for the GO-s9 suspension sonicated additional 10 min and by almost 40% for GO-s13 that were sonicated for 60 min longer than time sufficient to achieve maximum absorption coefficient ε_230nm_ = 4830 L g^−1^ m^−1^ (Fig. 7). An important observation is that too long ultrasound treatment have negative impact on the GO enhancement of hydrogen production. It is known that the use of sonication treatment exfoliates the multilayer GO flakes into individual GO sheets^44^. However, long-time sonication causes also extensive GO sheets fragmentation and defects (see Fig. 1, GO-s13)^45,46^. The amorphization can take place, however, we do not identify the nanometric scale carbon remnants, as well as the holes in GO structure. Thus, if they are present, they are supposed to have lateral sizes range of single nanometer or lower and thus below the microscopy (SEM and AFM) resolution. Therefore, we conclude that the decrease of the hydrogen production efficiency for the GO samples that underwent long sonication treatment is related to the decrease of the GO flakes size. To avoid negative impact of such fragmentation of GO flakes on the photocatalytic activity it is important to optimize the sonication treatment. Due to various bath sonicators used in the laboratories with different frequency, power or volume there is no unique sonication time that would ensure optimum GO properties for the photocatalytic activity. Thus, the correlation between the UV–Vis absorbance at 230 nm and the amount of H_2_ is the key, not the specific sonication parameters. Our work has shown that simple measurement of the optical spectra of the GO dispersion is reliable method for the optimization of the monolayer content. In short, the most important practical outcome of our results is that the optimum sonication time of the GO suspension used in photocatalytic H_2_ production systems should match exactly the time (but no longer) required to achieve the highest absorbance of the GO dispersion at 230 nm.

**Figure 8:**
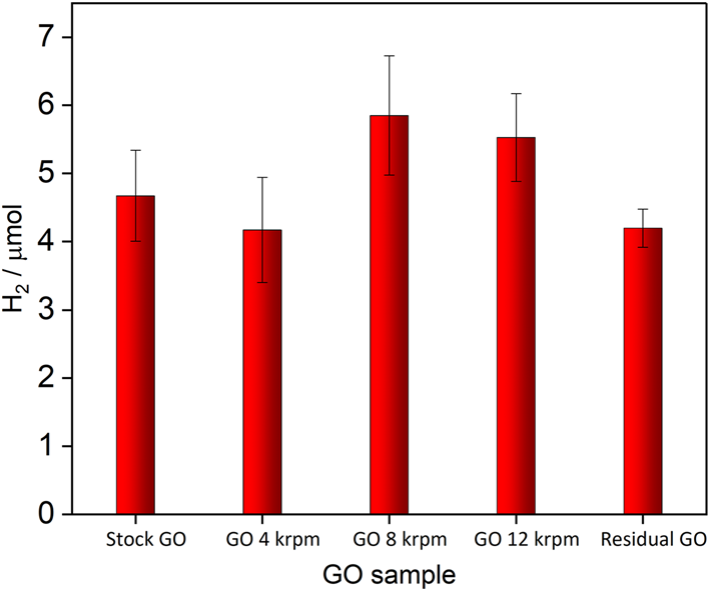
Amount of H_2_ evolved after 1 h of visible light irradiation on EY–GO–Co^2+^ system with various size-selected GO dispersions. Reaction conditions: EY (1 mM), GO (1.06 µg mL^-1^) TEOA (0.2 M), CoSO_4_ (2 × 10^−4^ M), pH = 10.8. The results are the average value and expanded uncertainty of four photocatalytic reactions, each conducted using independently prepared GO dispersion.

The investigation of the role of the sonication treatment of the GO suspension on the hydrogen production showed that photocatalytic efficiency is affected not only by the number of layers in GO flakes of the GO sample but as well as by the size of the flakes. To explore this effect in a quantitative manner the size-selected GO dispersion obtained via cascade centrifugation procedure were tested in the photocatalytic hydrogen production systems. The cascade centrifugation was performed four times and collected fractions were used independently for photocatalytic reaction with EY–GO–Co^2+^ system. The results are summarized in Fig. 8.

The highest efficiency of hydrogen generation was observed for GO-8 krpm which was higher by 22% in comparison to the analogue experiment with the “stock” GO. The small decrease of hydrogen production for GO-4 krpm might be attributed to the presence of graphene oxide aggregates as observed in AFM measurements. For the residual GO the decrease of the hydrogen production by 30% was observed in comparison to GO-8 krpm sample. The residual GO consists mostly of monolayers but with significantly smaller flakes size than fraction GO-8krpm (Fig. 5). The EY–GO–Co^2+^ system produces the highest amount of H_2_ when graphene oxide occurs as monolayers and efficiency becomes lower with decrease of GO sheets size. It is important to choose appropriate conditions of sonication process to obtain GO in optimal form as monolayered flakes with the biggest, possible area. The influence of the GO number layers on the hydrogen production can be related to the change of surface area which subsequently may affect the interaction with precatalyst Co^2+^. The interaction of GO and EY was found previously to be weak and therefore we do not expect to be dependent on GO morphology^25^. Therefore, presumably, the change in the size and dispersion of Co nanoparticles on the GO surface is responsible for the observed changes of hydrogen production upon variation of GO morphology. Detailed analysis of the dependence of Co nanoparticles size and dispersion on GO morphology will be the subject of separate paper in the future.

## Conclusions

This work provides an important guide for researchers interested in exploiting the application of graphene-based materials in photocatalytic water splitting. We report a systematic and comprehensive study of the influence of the morphology of graphene oxide flakes on their photocatalytic activity toward hydrogen production. This information is absolutely crucial given the interest in using graphene-based materials in photocatalytic water splitting.

Therefore our systematic exploration of the effect of the GO size and the number of layers provide a guide for optimizing the photocatalytic hydrogen production efficiency with graphene-based materials. Our results clearly demonstrate that both size and the number of layers of GO flakes do matter for the photocatalytic hydrogen production, but the latter one has more profound impact on the hydrogen production efficiency. Our results demonstrate that sonication of graphene oxide is an important contributor to the variation in efficiency of hydrogen production with GO-based composites. With this concept in mind, it is therefore worthwhile for researchers to control and optimize the sonication time of GO prior to preparation of GO-based materials. Our observed effect of the morphology of GO dispersions on photocatalytic efficiency could be employed for other 2D-material based composites with various potential application.
